# The production and application of carbon nanomaterials from high alkali silicate herbaceous biomass

**DOI:** 10.1038/s41598-020-59481-7

**Published:** 2020-02-13

**Authors:** Ahmed I. Osman, Charlie Farrell, Ala’a H. Al-Muhtaseb, John Harrison, David W. Rooney

**Affiliations:** 1 School of Chemistry and Chemical Engineering, Queen’s University Belfast, Belfast, BT9 5AG Northern Ireland UK; 2 Chemistry Department, Faculty of Science - Qena, South Valley University, Qena, 83523 Egypt; 3 South West College, Cookstown, Co, Tyrone, BT80 8DN Northern Ireland UK; 4 School of Mechanical and Aerospace Engineering, Queen’s University Belfast, Belfast, BT9 5AH Northern Ireland UK; 5 Department of Petroleum and Chemical Engineering, College of Engineering, Sultan Qaboos University, Muscat, Oman

**Keywords:** Pollution remediation, Nanoscale materials

## Abstract

Herein, value-added materials such as activated carbon and carbon nanotubes were synthesized from low-value *Miscanthus* × *giganteus* lignocellulosic biomass. A significant drawback of using *Miscanthus* in an energy application is the melting during the combustion due to its high alkali silicate content. An application of an alternative approach was proposed herein for synthesis of activated carbon from *Miscanthus* × *giganteus*, where the produced activated carbon possessed a high surface area and pore volume of 0.92 cm^3^.g^−1^ after two activation steps using phosphoric acid and potassium hydroxide. The S_BET_ of the raw biomass, after first activation and second activation methods showed 17, 1142 and 1368 m^2^.g^−1^, respectively. Transforming this otherwise waste material into a useful product where its material properties can be utilized is an example of promoting the circular economy by valorising waste lignocellulosic biomass to widely sought-after high surface area activated carbon and subsequently, unconventional multi-walled carbon nanotubes. This was achieved when the activated carbon produced was mixed with nitrogen-based material and iron precursor, where it produced hydrophilic multi-wall carbon nanotubes with a contact angle of θ = 9.88°, compared to the raw biomass. synthesised materials were tested in heavy metal removal tests using a lead solution, where the maximum lead absorption was observed for sample AC-K, with a 90% removal capacity after the first hour of testing. The synthesis of these up-cycled materials can have potential opportunities in the areas of wastewater treatment or other activated carbon/carbon nanotube end uses with a rapid cycle time.

## Introduction

To date, most of the global energy demand is facilitated through the continued use of fossil fuels and there is undoubtedly a growing interest in finding and promoting alternatives such as fuels with low CO_2_ and particulate emissions by switching from non-renewable to renewable natural resources. One of the most promising solutions widely reported is to use biomass as an energy crop due to its carbon neutrality. For instance, biomass has been utilised in combined heat and power systems (CHP) to generate electricity/heat or through gasification to convert it into syngas (a mixture of CO + H_2_), which possesses a high net calorific value and could be used for power generation. Considering other thermochemical conversion methods such as combustion, however, can encounter certain problems when using problematic feedstocks such as miscanthus which produces a high amount of silica ash that melts and clogs the furnaces as part of normal incineration boilers along with the emissions (NOx, SOx and particulates). This can ultimately lead to significant downtimes in combustion processes due to the fouling/clogging and is not ideal at any scale. The emissions associated are accounted for and allocated in three fundamental categories; human health, energy resources and ecosystem quality^1^. This can be avoided by introducing oxygen-free/inert methods such as pyrolysis, which leads to a combination of multiple useful products in various phases that contribute positively to the circular economy via up-cycling and creation of added value products^2^. One of the well-known strategies of cleaner production practitioners is to increase production efficiency and reduce waste and emissions^3^. Interestingly, biomass can be utilised more than just a fuel, whereby it can be converted into activated carbon (AC) that has been extensively used as a remediation solution for wastewater streams polluted with heavy metals. Thus, converting biomass waste into valuable products such as this will help to achieve two of the seventeen Sustainable Development Goals (SDGs) outlined by the united nations; affordable and clean energy along with clean water and sanitation^4^. AC has the potential to be a powerful adsorbent material due to its high porosity, additionally to possessing high surface area. Steinmann *et al*.^5^ stated that there are two particularly important qualitative aspects of recycling: the quality of the recycled material (recyclate) and the functionality post recycling process. The assessment and evaluation the of the energy, along with monitoring the related emissions witihn the recycling process should be taken into consideration as well^6–8^. Therefore, considering AC as a powerful tool in facilitating and achieving a circular economy by providing end uses or other routes in the lifecycle of waste biomass sources, minimising waste and promoting sustainable development due to its valorisation and the substantial increase in surface area and porosity from the up-cycling^9^. AC can also be seen as providing added value to otherwise problematic waste materials and thus up-cycling in this particular area helps with using this feedstock for thermochemical conversion by providing an alternate pathway.

Extensive research currently exists on the preparation of porous high-surface-area ACs from lignocellulosic biomass along with their potential application in eliminating pollutants by adsorbing molecules from both the gas and liquid phase^10–14^. There are two common ways in the activation of biomass: physical and chemical activation, while the latter is more commonly used. The former involves the carbonisation of biomass in an inert atmosphere such as CO_2_ in the temperature range of 800–1000 °C. The latter, in turn, involves the impregnation of the biomass with chemicals, for instance, H_3_PO_4_, KOH or ZnCl_2_. Jadwiga *et al*. prepared ACs materials of high surface area from miscanthus grass, walnut shells and energetic willow and wheat straw along with a characterisation of their resultant AC adsorption properties^15^. In this particular study, they used a physical activation method using CO_2_, H_2_O (steam) or microwave energy. The surface areas of ACs that were derived from miscanthus were in the range of (429–748 m^2^.g^−1^). Michel *et al*.^16^, also used *miscanthus* × *giganteus* to synthesise ACs with surface areas as high as 800–900 m^2^.g^−1^ via a pyrolysis process using a tubular fixed-bed reactor and rotatory kiln.

One of the advantages of using chemical activation over the physical activation method is the ability to synthesise ACs with a porous structure along with producing higher surface area materials. Yorgun *et al*.^10^, prepared a high surface area (2736 m^2^.g^−1^) ACs from Paulownia wood using ZnCl_2_ activation. Kasperiski *et al*. prepared ACs with surface areas (1480 m^2^.g^−1^) from *Caesalpinia ferrea* seed pod wastes using ZnCl_2_ with different biomass: ZnCl_2_ weight ratios of (1: 0.5, 1:1 and 1:1.5)^17^. However, compared with other potential activating agents (such as H_3_PO_4_ and KOH), ZnCl_2_ is considered to be the most expensive activating agent^10^. On the other hand, the cheapest activating agent is phosphoric acid, and it has been extensively used in research in this area. For instance, evergreen oak was used as a raw material to prepare ACs using different concentrations of H_3_PO_4_ and pyrolysis temperatures^18^. Wherein, the optimum condition was determined to be 60% H_3_PO_4_ concentration along with pyrolysis temperature of 450 °C to produce ACs with a surface area of 1723 m^2^.g^−1^ ^11^. Alau *et al*.^19^, investigated the preparation of ACs from Neem husk using H_3_PO_4_, KOH and ZnCl_2_ as activating agents with the optimum activating temperatures at 500, 350 and 400 °C, respectively. The ACs prepared from jatropha wood activated by KOH gave higher surface areas than that of H_3_PO_4_ with S_BET_ of 1305 and 751 m^2^.g^−1^, respectively^20^. Interestingly, a multi-step activation process using H_3_PO_4_ followed by KOH produced ACs of high surface area of 2595 m^2^.g^−1^ ^21^. Thus, it would appear that multi-step activation showed significantly better results compared to the single-step activation in terms of porosity and surface area. Additionally, miscanthus pyrolysis into biocarbon was improved through the synergistic catalytic effect of Fe^3+^ and Co^2+^ during the pyrolysis process at a temperature of 900 °C^22^.

ACs also provide a pathway towards the production of Carbon Nanotubes (CNTs), which has arguably a larger potential than AC due to their strength comparable to steel, and their electrical and thermal conductivity properties make them highly versatile and a perfect candidate for many industrial applications^23^. CNTs can be produced by different methods such as Chemical Vapour Deposition (CVD), Laser Ablation, Arc Discharge along with the pyrolysis of lignocellulosic biomass. There are limited instances within the literature that utilise ACs that are derived from biomass to produce CNTs. Yao *et al*.^24^ prepared CNTs from AC derived from the pyrolysis of waste biomass using melamine as a nitrogen-based compound along with ferrous sulphate as an iron source. The AC produced possessed a surface area of 1604 m^2^.g^−1^ and was consequently used to prepare the CNTs materials with a surface area in the range of 614–952 m^2^.g^−1^.

This study proposes a method of up-cycling waste lignocellulosic biomass in the form of *miscanthus* × *giganteus* by synthesising AC and CNTs which can have a dual-role in this given waste stream. The AC and CNTs can remove heavy metal ions from wastewater sources and using this type of waste biomass in this way can remove the potentially problematic high melting alkali silicates that are encountered when using this particular resource for thermochemical conversions such as combustion or pyrolysis. Another potential impact of this research is that the CNTs produced are of a hydrophilic nature, whereas conventional CNTs from biomass sources are usually hydrophobic. To the best of the author’s knowledge, this is the first detailed study of preparing high surface area AC and then further, hydrophilic multi-walled hydrophilic CNTs from waste *miscanthus* × *giganteus*.

## Results and Discussion of Lignocellulosic Biomass Characterisation

**Figure 1:**
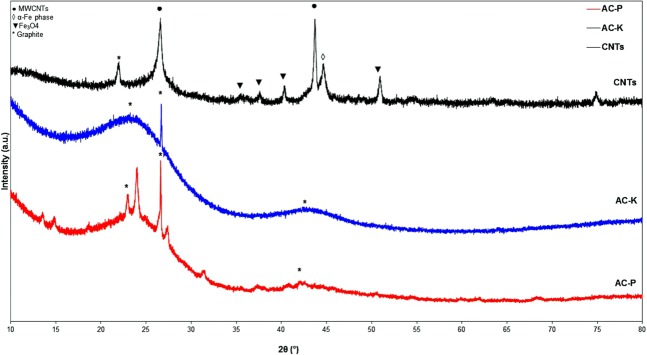
XRD patterns of samples of activated carbon using firstly phosphoric acid (AC-P) and secondly using potassium hydroxide (KOH) along with the Carbon nanotubes (CNTs).

The XRD diffractogram patterns of the activated carbons along with the carbon nanotubes are shown in Fig. 1. Both the AC samples show mainly two diffraction peaks in the 2θ range of 24.9 and 42.9°, which corresponds to different types of crystallite graphite, however, AC-P seems to have impurities from phosphoric acid or incomplete activation as most of those diffraction peaks disappeared in the second activation i.e. in AC-K sample. The CNTs sample shows the two diffraction peaks characterised for the Multi-wall Carbon nanotubes (MWCNTs) at 2θ of 26.6° (002 plane) which also could be observed in hexagonal graphite along with the diffraction peak at 43.6 (100 plane)^25^. Iron species in the CNTs show two types of diffraction peaks; Fe_3_O_4_ at 2θ of 35.9, 37.3 40.4, 43.6 and 50.7° and α-Fe phase at 2θ of 44.7° ^24^.

**Figure 2:**
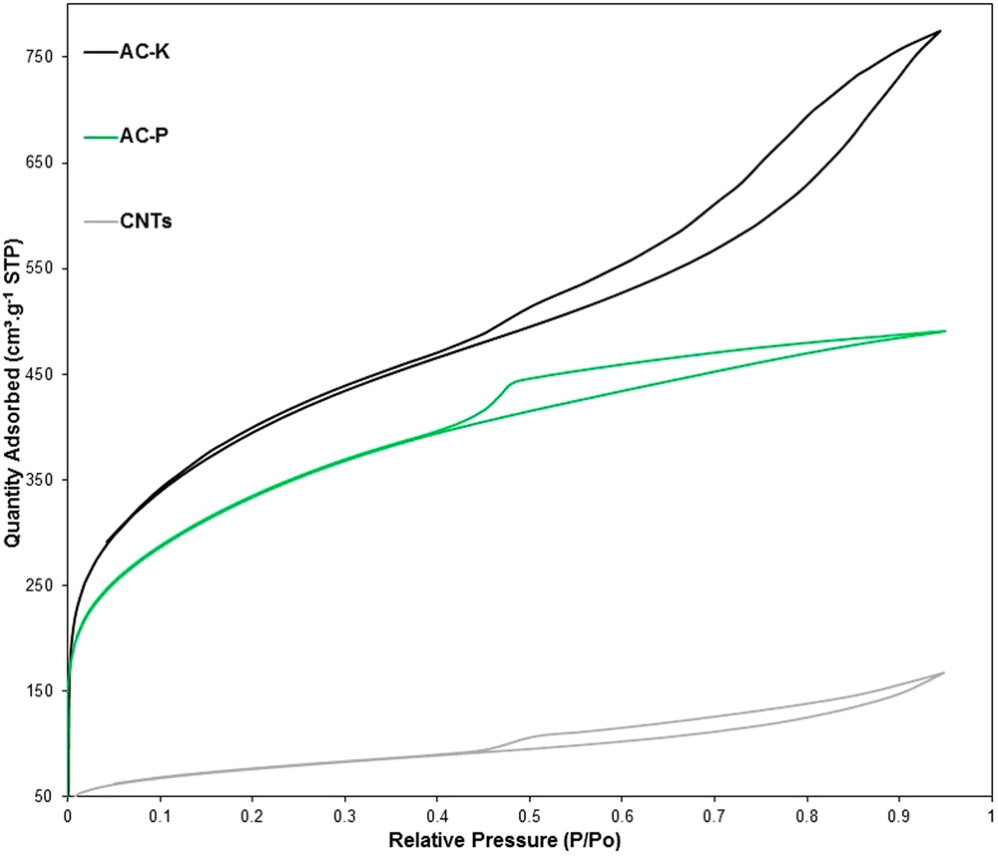
Nitrogen adsorption-desorption isotherms at 77 K of samples of activated carbon using firstly phosphoric acid (AC-P) and secondly using potassium hydroxide (KOH) along with the Carbon nanotubes (CNTs).

Initially, the DMP sample offered a low surface area of 17 m^2^.g^−1^ which was significantly enhanced through the first activation using phosphoric acid (AC-P) to reach 1142 m^2^.g^−1^ and further increased with the second activation (AC-K) to 1368 m^2^.g^−1^ as shown in Table 1. The adsorption/desorption isotherms in all samples are of IV type in the relative pressure range of 0.4–0.9, implying the formation of mesoporous carbonaceous materials, which is in line with the work of Morali *et al*.^26^. Moreover, the pore volume also significantly increased from 0.0018 to 0.70 and 0.92 cm^3^.g^−1^, for the DMP, AC-P and AC-K samples, respectively as seen in Table 1 and from the N_2_ adsorption-desorption isotherms shown in Fig. 2. The increase in the surface area and the pore volume is attributed to the decline of H, O and N contents in miscanthus during the activation process along with the formation of porous carbon material^14^. On the other hand, the CNTs showed a surface area of 260 m^2^.g^−1^ and a pore volume of 0.17 cm^3^.g^−1^. The surface of CNTs varies depending on the wall thickness of the CNTs produced. For instance, the maximum theoretical prediction of the surface area of a single wall CNTs (SWCNTs) is 1375 m^2^.g^−1^, while MWCNTs are characterised by surface area ranging from 15–500 m^2^.g^−1^. Where a surface area of 50, 175 and 500 m^2^.g^−1^ corresponds to 40-walled (35 nm diameter), 10-walled (15 nm diameter) and 3-walled (6 nm in diameter) MWCNT. Given the surface area reported in this study, it is obvious that the produced CNTs are MWCTs with multiple walls of 3–10 walls and 6–15 nm diameter^27^. It is worth noting that the lower surface area of the CNTs could be due to the presence of amorphous carbon particles, impurities, multilayer polygonal particles and large graphite platelets^28^. Interestingly, the formation of MWCNTs has significant advantages over SWCNTs due to its enhanced chemical, low product cost per unit and thermal stability^29^.

**Table 1:** Physicochemical characterisations of miscanthus dry plant, AC-H_3_PO_4_, AC-KOH and CNTs samples.

Samples			DMP	AC- P	AC-K	CNTs
Elemental composition *(wt.% on dry basis)*		% C	42.85	69.82	72.66	70.9
		% H	5.83	2.07	1.24	0.3
		% N	1.21	0.3	0.3	3.42
		% S	0.1	0.1	0.1	0.1
		% O	50.01	27.71	25.70	25.28
EDX analysis *(wt.% on dry basis)*		C	61.7	79.5	82.8	85.3
		O	35.8	17.0	17.1	5.6
		K	0.9	—	—	—
		Si	0.6	0.5	—	—
		Ca	0.3	—	—	—
		P	0.1	2.7	—	—
		Fe	—	—	—	7.2
		S	0.2	0.3	—	0.6
S_BET_ results	S_BET_(m^2^.g^−1^)		17	1142	1368	260
	Pore volume (cm^3^.g^−1^)		0.0018	0.70	0.92	0.17
	Pore size (*A°*)*		18.9	20.3	40.1	47.8

**Figure 3:**
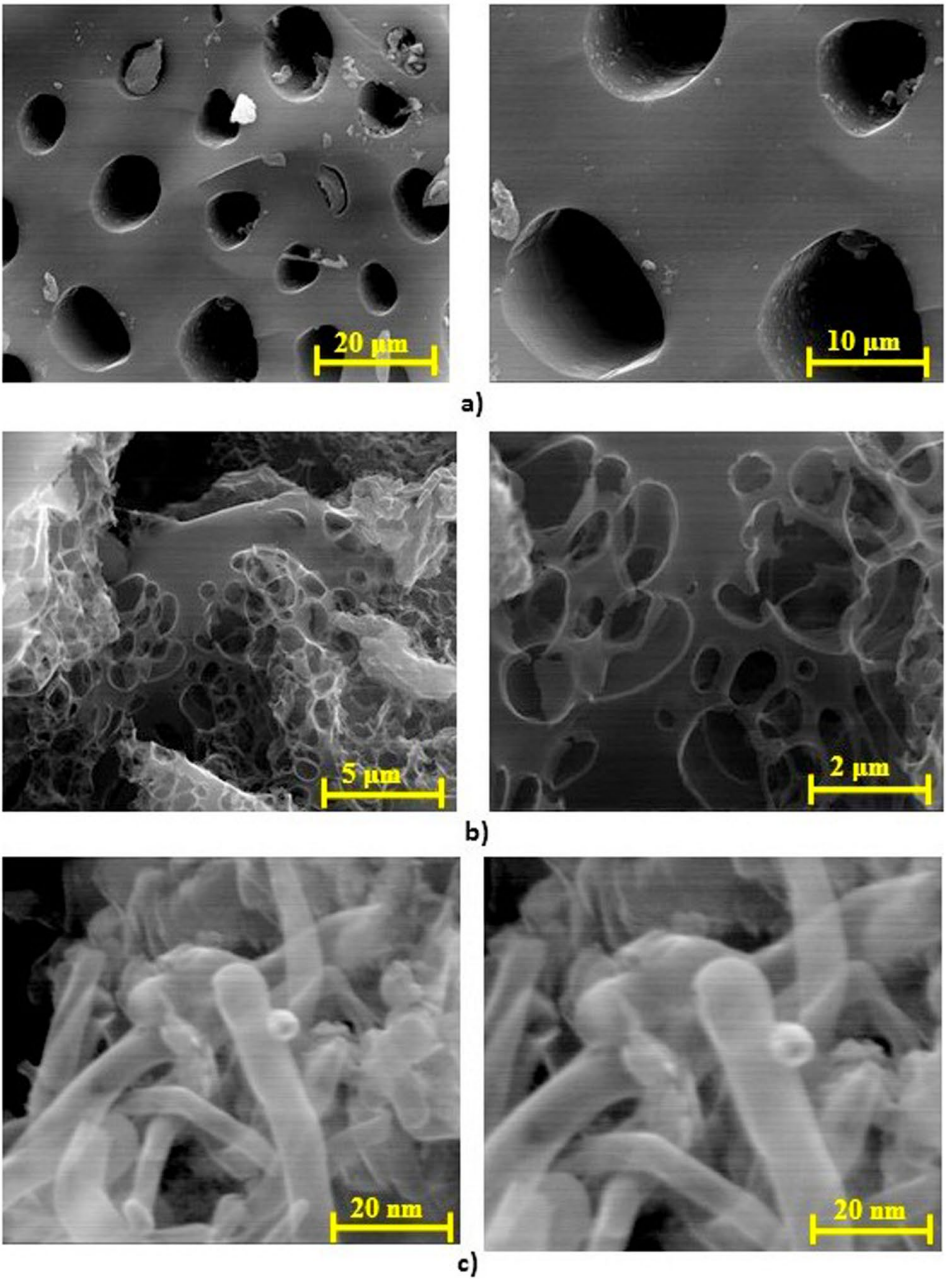
SEM images for (**a**) H_3_PO_4_ activation (AC-P), (**b**) KOH activation (AC-K) and (**c**) CNTs at different levels of magnification using ETD detector.

The DMP showed a typical lignocellulosic biomass composition of 41–45 wt.% C, 5.7–5.9 wt.% H, 1.2–1.8 wt.% N and 47–50 wt.% O along with a minor contribution of sulphur ~0.2 wt.%, this is in agreement with the previous publication^30^. The SEM images of the DMP in previous work showed large fibrils arranged in the fascicular texture form^30^. Figure 3 shows the SEM images of the ACs either with phosphoric acid (AC-P) or potassium hydroxide (AC-K) along with the CNTs at a different level of magnifications using the ETD detector. Mixing the DMP with phosphoric acid in the first activation, followed by the pyrolysis process has created a porous carbon as shown in Fig. 3(a). Thus, this reflects on the surface area; which was significantly enhanced as confirmed by the S_BET_ results, along with the pore volume which increased from 0.0018 to 0.70 cm^3^.g^−1^ for the first activation (AC-P). With further activation using KOH, the formation of more channelling pores with multilayer formation within the AC-K as shown in Fig. 3(b), which is in agreement with the S_BET_ that showed an enhancement in the surface area and the pore volume as well; as shown in Table 1. Figure 3(c) shows the SEM images of the CNTs which clearly showed the formation of carbon nanotubes. In order to observe the carbon nanotubes, a high magnification (725,000×) was required in comparison with what was used for the activated carbon (10,000×).

The EDX analysis for the produced ACs and the CNTs is shown in Table 1 and Fig. S1. It is well known that SEM-EDX analysis is a surface technique which gives the elemental composition in wt.%. The wt.% of C increased by 17.8% from the DMP to the AC-P sample, this is due to the decomposition of the lignocellulosic compounds (cellulose, hemicellulose and lignin) along with the evolution of the phenolic gases due to the decomposition of lignin and the formation of porous carbon materials. Interestingly, most of the inorganic elements in the DMP disappeared by the formation of ACs such as K, Ca and Fe as shown in Table 1. AC-P showed the presence of 2.7 wt.% of P which is due to using phosphoric acid in the first activation process. In the second activation step, the carbon percentage increased to 82.8 wt.%. The CNTs sample showed a 7.2 wt.% of Fe which is due to using iron oxalate (Fe_2_(C_2_O_4_)_3_ 6H_2_O during the preparation of CNTs.

**Figure 4:**
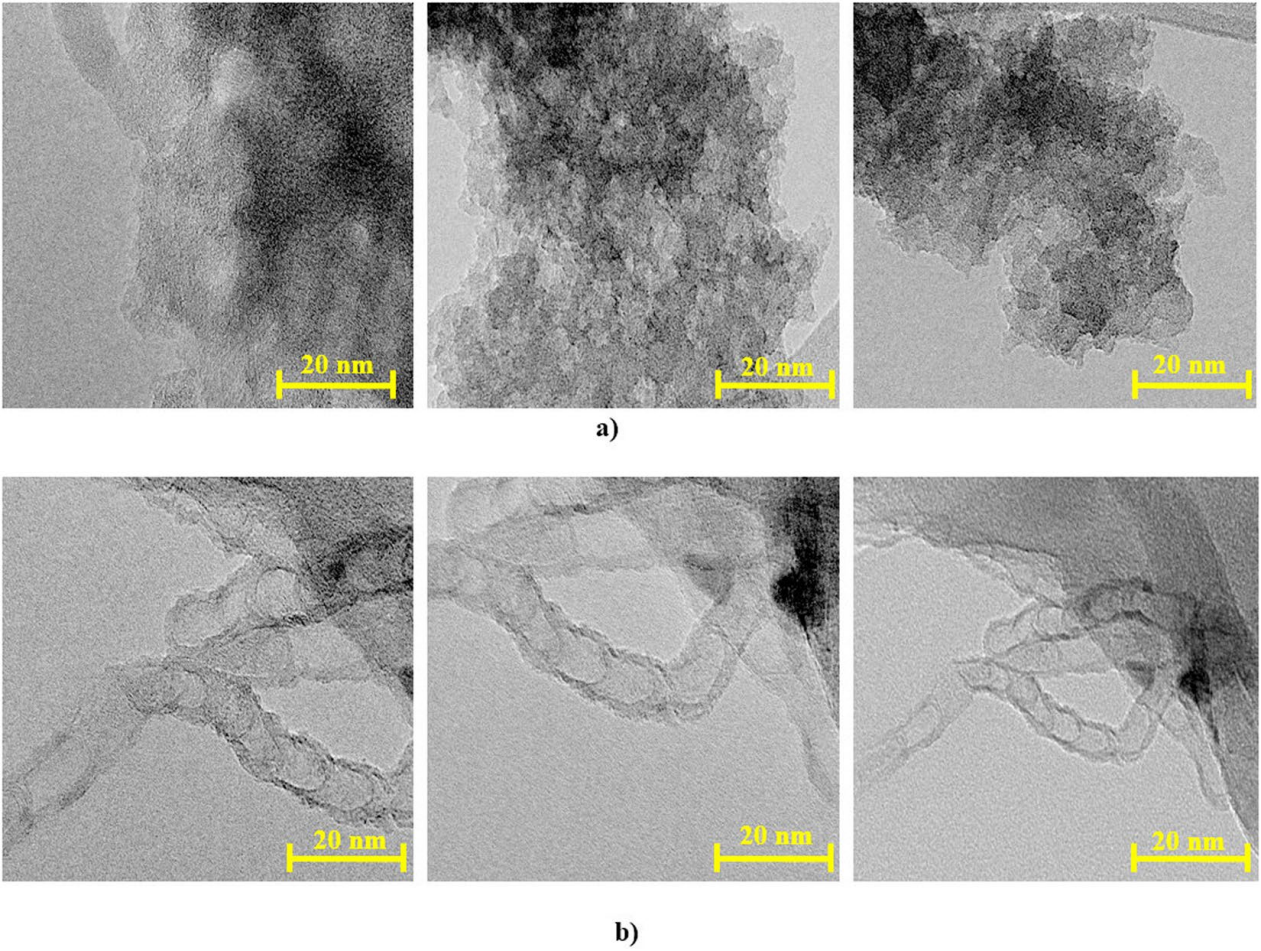
TEM images for (**a**) activated carbon (AC-K) and (**b**) CNTs.

The TEM images of the activated carbon (AC-K) along with the carbon nanotubes (CNTs) are shown in Fig. 4a,b, respectively. The carbon atoms in the AC-K sample are porous which is in agreement with the S_BET_ results in Fig. 2. The carbon nanotubes channels in Fig. 4(b) are in the range of 9–11 nm which is in agreement with the results derived from the adsorption/desorption nitrogen curves that showed the formation of MWCTs with a multiwalled of 3–10 walls and 6–15 nm diameter^27^.

**Figure 5:**
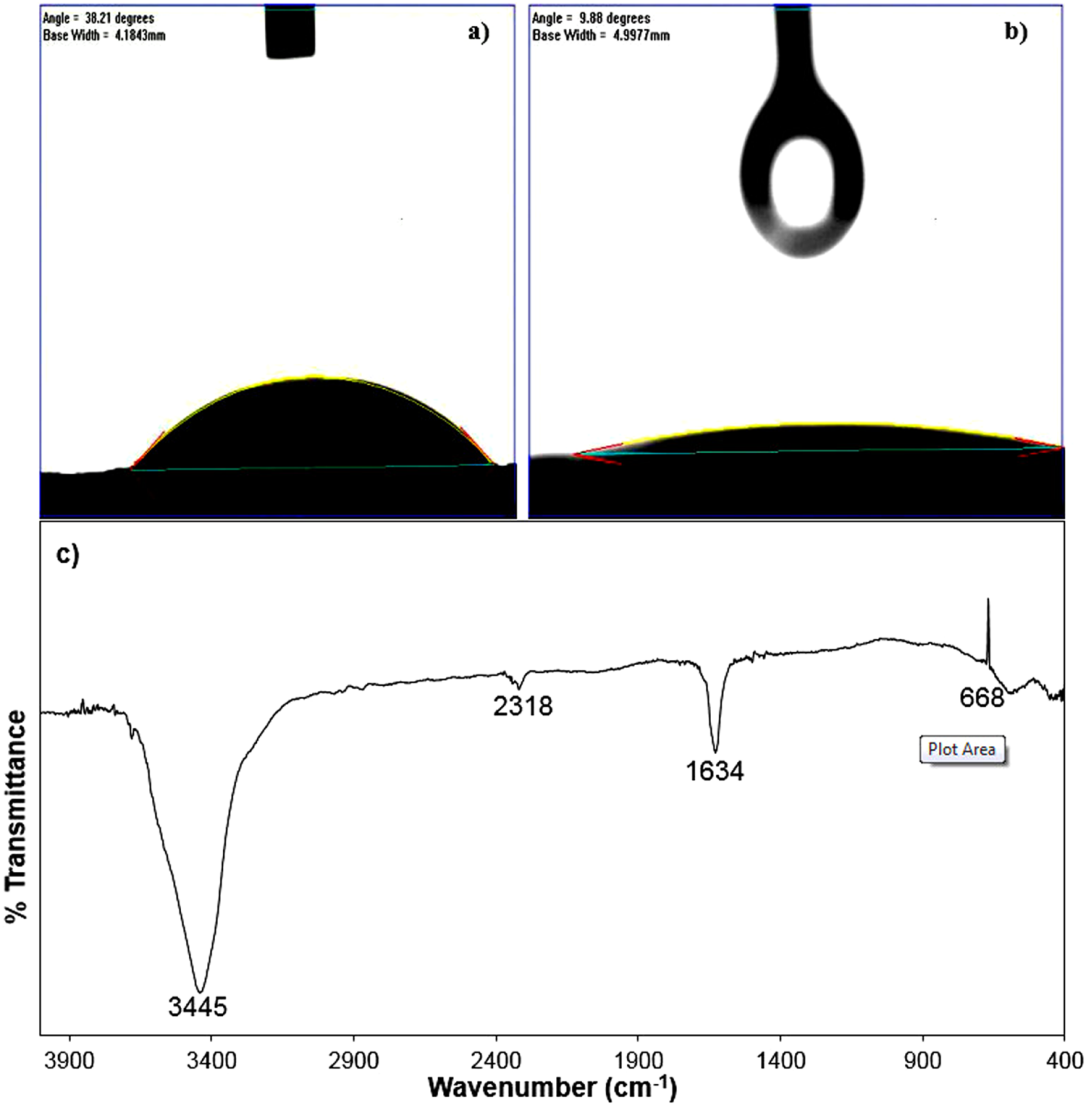
The water contact angle analysis of DMP (**a**) and the produced CNTs (**b**) along with the FT-IR of the CNTs (**c**).

The water contact angle analysis was conducted to determine the hydrophilicity of the DMP and final produced CNTs as shown in Fig. 5. The wettability of either the lignocellulosic biomass or CNTs exhibited by the contact angle in θ of the water droplet is assumed by Young’s equation (Eq. 1)

1 $$\cos \,\theta =\frac{{\gamma }_{SV}\,-{\gamma }_{SL}}{{\gamma }_{LV}}$$

where, *γ*_*SV*_, *γ*_*SL*_ and *γ*_*LV*_ stand for the interfacial surface tension of solid (S), liquid (L) and gas vapour (V). The surface hydrophilicity is calculated according to the water contact angle and is divided into four different categories; super-hydrophilic (θ < 10°), hydrophilic (10 < θ < 90°), hydrophobic (90 < θ < 150°) and super-hydrophobic (θ > 150°). The DMP showed a contact angle of θ = 38.21°, thus the dry lignocellulosic biomass of miscanthus was hydrophilic as seen in Fig. 5(a). According to the preparation method used in this study, the final CNTs produced offered a super-hydrophilic surface with a contact angle of θ = 9.88° as shown in Fig. 5(b). The results are in agreement with the work of Janas and Stando who managed to prepare a super-hydrophilic CNTs with a contact angle of 7° ^31^. Min *et al*.^32^ also prepared SWCNTs with a water contact angle of nearly 0°.

The FT-IR of the CNTs in the wavenumber of 400–4000 cm^−1^ is shown in Fig. 5(c). The broadband at 3445 cm^−1^ is attributed to the stretching of the OH group, which is related to the associated water and the stretching of the carbon nanotubes backbone^33,34^. The band at 2318 cm^−1^ is ascribed to the C≡N stretching and this is might be due to the nitrogen bonding in the CNTs from the melamine used in the study. Moreover, the band at 1634 cm^−1^ is attributed to the aromatic like C=C stretching mode of the MWCNTs graphitic layers or carbonyl groups at 1626 cm^−1^ ^33^. The broad water band at 3445 cm^−1^ showed that the produced CNTs is hydrophilic which is in agreement with the results of water angle results shown in Fig. 5(b). Usually, the produced CNTs typically presents hydrophobic interfaces. For instance, Alnarabiji *et al*.^35^ prepared a hydrophobic MWCNTs with a contact angle of θ = 136°. Lau *et al*.^36^ even reported a water angle of θ = 161° for a superhydrophobic CNTs. Again, CNTs in nature are either hydrophobic or superhydrophobic, unless treated with functional groups in order to prepare hydrophilic CNTs. Herein, according to our preparation method which included two activation steps along with the addition of melamine and iron-based precursor, the produced CNTs was superhydrophilic MWCNTs, which have more advantages over the hydrophobic CNTs. In water adsorption applications, water vapour is rarely adsorbed in the hydrophobic CNTs at low pressure, while, hydrophilic CNTs adsorb the water vapour at low pressure^37^. Ohba *et al*.^37^ investigated changing the water affinity in hydrophobic CNTs into hydrophilic CNTs and it was found to be dependant on the channel size and the adsorption-desorption specific process.

The carbon nanotubes produced herein can be used for several industrial applications such as microelectronics, smart & novel composites, bone growth in tissue engineering and wastewater remediation. For instance, carbon nanotubes have significance and are widely sought after in the micro-electronics application as they can be used to make transistors and their performance has been proven to outperform that of the conventional silicon semiconductor used in microelectronics. CNTs electrical conductivity has been reported to be 100 times higher than silicon semiconductor when voltage is applied^38^. They can operate faster, more efficient and at a lower voltage generating less heat. The geometry and size of the CNTs mean that more transistors made from this material can be placed on a chip that what is currently done with silicon. The hydrophilic nature of the carbon nanotubes produced in this study increases the applicability of this material to be used in electrical storage applications either in the form of a transistor or a battery and therefore, has two major added benefits from this type of industrial application.

Also, the fact that carbon nanotubes are 100% natural carbon, means that they are compatible with cells and organic matter. The fact that it is hydrophilic would effectively mimic the nature of a cell taking in water via osmosis. This means that any CNT material used for bone scaffolding will not have any cytotoxic effects on a patient or the human body. This again, in itself is another industrial-scale application that the carbon nanotubes created in this study could be used for.

**Figure 6:**
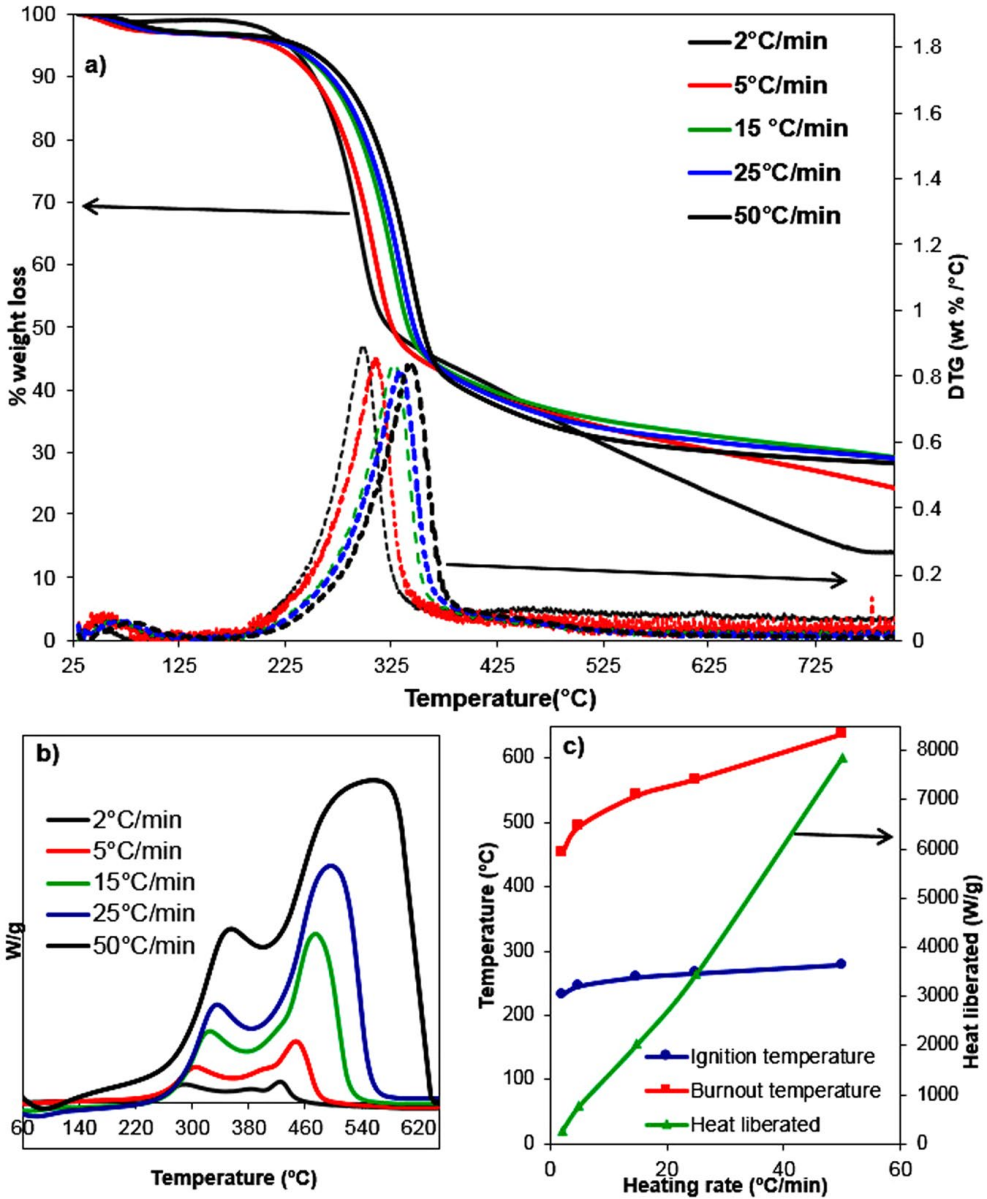
Thermal analysis of DMP (**a**) TGA-DTG curves under the N_2_ atmosphere, (**b**) DSC curves with different heating rates under air atmosphere along with (**c**) the calculated ignition, burnout temperatures and heat liberated during the combustion of DMP.

The TGA-DTG (5a) and DSC (5b) curves of DMP under nitrogen and air atmosphere are shown in Fig. 6, respectively. Miscanthus pyrolysis showed an average of 70–80% weight loss as shown in Fig. 6(a) which is in agreement with the previous work (72.5%)^30^. DTG curves, Fig. 6(a), show the rate of the % weight loss with the highest rate found at a temperature of 293, 311, 327, 335 and 343 °C at a heating rate of 2, 5, 15, 25 and 50 °C.min^−1^. It is not surprising that the maximum peak of weight loss shifted toward the higher pyrolysis temperature by 50 °C with increasing the heating rate from 2 to 50 °C.min^−1^. The DMP combustion is shown in Fig. 6(b) with two combustion stages at temperature ranges of 225–398 °C and 412–637 °C. Again, by increasing the heating rate, the two combustion peaks were shifted toward higher temperatures. Table 2 shows the calculated ignition and burnout temperatures along with the heat released by the combustion of DMP, which all increased with different trends by increasing the heating rates from 2 °C.min^−1^ to 50 °C.min^−1^. Interestingly, the ignition temperatures to some extent increased by only 45 °C, while the burnout temperature increased by 184 °C as shown in Fig. 6(c). On the other hand, the heat liberated (W.g^−1^) during the combustion of DMP significantly increased by more than thirty times with increasing the heating rates by the same value from around 250 to 7867 W.g^−1^ as shown in Fig. 6(c) and Table 2. The TGA curve of the CNTs (Fig. S2) shows 16% weight loss in the temperature range 50–1000 °C, this is in agreement with the work done by He *et al*.^39^, who reported a similar weight loss of approximately 20 wt.% along with Guan *et al*.^40^, with a weight loss of 18 wt.%.

**Table 2:** Calculated ignition and burnout temperatures along with the heat liberated during the combustion of miscanthus under air atmosphere.

Heating rate	Ignition temperature (°C)	Burnout temperature (°C)	Heat liberated (W.g^−1^)
2 °C.min^−1^	233	453	250
5 °C.min^−1^	246	494	766
15 °C.min^−1^	259	542	2034
25 °C.min^−1^	265	567	3451
50 °C.min^−1^	278	637	7867

The XPS analysis of the activated carbon (AC-P) along with the carbon nanotubes (CNTs) are shown in Fig. S3(a–g) along with the composition obtained given in Table 3. The binding energy of carbon atoms slightly increased from 284.4 to 284.6 eV for AC-P and CNTs, respectively. On the other hand, the binding energies of O*1s* shifted toward lower binding energies from 532.9 to 532.6 eV for AC-P and CNTs, respectively as seen in Fig. S3(b), implying that the oxide species decreased during the formation of carbon nanotubes which is confirmed by the dramatic decrease in the atomic weight percentage (at.wt.%) of 18.2 and 7.9 at.wt.% for AC-P and CNTs, respectively. It is not surprising that the at.wt.% for N*1s* significantly increased for AC-P and CNTs of 0.8 and 7.8 at.wt.%, respectively due to the presence of melamine as a nitrogen source during the preparation of CNTs as seen in Fig. S3(c). Also, the presence of Fe*2p* is due to iron oxalate which was used as an iron source during the preparation of CNTs as seen in Fig. S3(f). On the other hand, the P*2p* peak disappeared during the transformation of activated carbon into CNTs as seen in Figure S3(e). It is well known that during the thermochemical decomposition of miscanthus tends to form silicates compounds, thus the Si*2p* slightly increased from 1.5 to 2.1 at.wt% for AC-P and CNTs, respectively. The XPS survey (Fig. S3(g)) for both of AC-P and CNTs looks similar apart from the dramatic increase in the N*1s*, the appearance of an extra peak of Fe*2p* along with the absence of P*2p* during the transformation of activated carbon into CNTs.

**Table 3:** XPS results of the activated carbon AC-P along with the CNTs sample.

SAMPLE	C*1s*		O*1s*		N*1s*		Si*2p*		P*2p*		Fe*2p*		Na*1s*	
	Peak B.E.	Atomic wt.%	Peak B.E.	Atomic wt.%	Peak B.E.	Atomic wt.%	Peak B.E.	Atomic wt.%	Peak B.E.	Atomic wt.%	Peak B.E.	Atomic wt.%	Peak B.E.	Atomic wt.%
AC-P	284.4	75.6	532.9	18.2	400.6	0.8	104.1	1.5	134.8	3.7	—	—	1072.6	0.05
CNTS	284.6	81.5	532.6	7.9	398.7	7.8	103.4	2.1	—	—	711.1	0.7	—	—

### Heavy metal removal application

**Figure 7:**
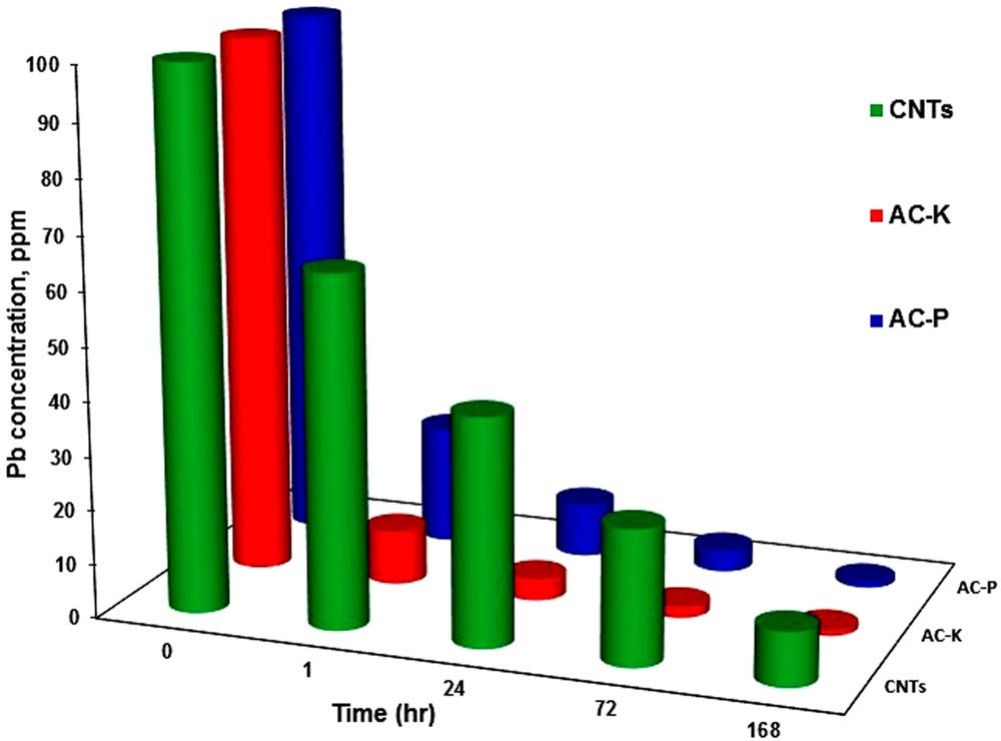
The heavy metal removal test of lead on AC-P, AC-K along with the CNTs in a 168 hour test.

Figure 7 shows the lead removal capacities of AC-P, AC-K and CNTs, where the observed activity was as the following series; AC-K > AC-P > CNTs. After one hour of the test, AC-K showed 90% removal while AC-P and CNTs showed 78, and 35%, respectively. The lowest HMR of the CNTs may be due to the lower surface area along with the pore volume. It is not surprising that AC-P showed lower HMR than that of AC-K as it showed a lower surface area as shown in Table 1. Interestingly, AC-P showed 83.4% of the HMR value of AC-K and at the same time it showed 86% of its surface area (S_BET_ AC-P/S_BET_ AC-K) * 100)). Thus, implying that the surface area plays a crucial role in the HMR herein. At 72 hrs HMR test, the AC-K, AC-P and CNTs showed 98, 96 and 75% HMR. While, at 168 hrs test, both AC-K and AC-P removed almost all the lead metal with the activity of 99%, whilst CNTs showed only 75% HMR as shown in Fig. 7. This means that the first activation method using phosphoric acid improved the morphology of the produced activated carbon (AC-P) by creating new pores on the surface. Furthermore, the second activation method using KOH showed further improvement in surface area and the morphology which resulted in achieving the highest heavy metal removal in this study.

**Figure 8:**
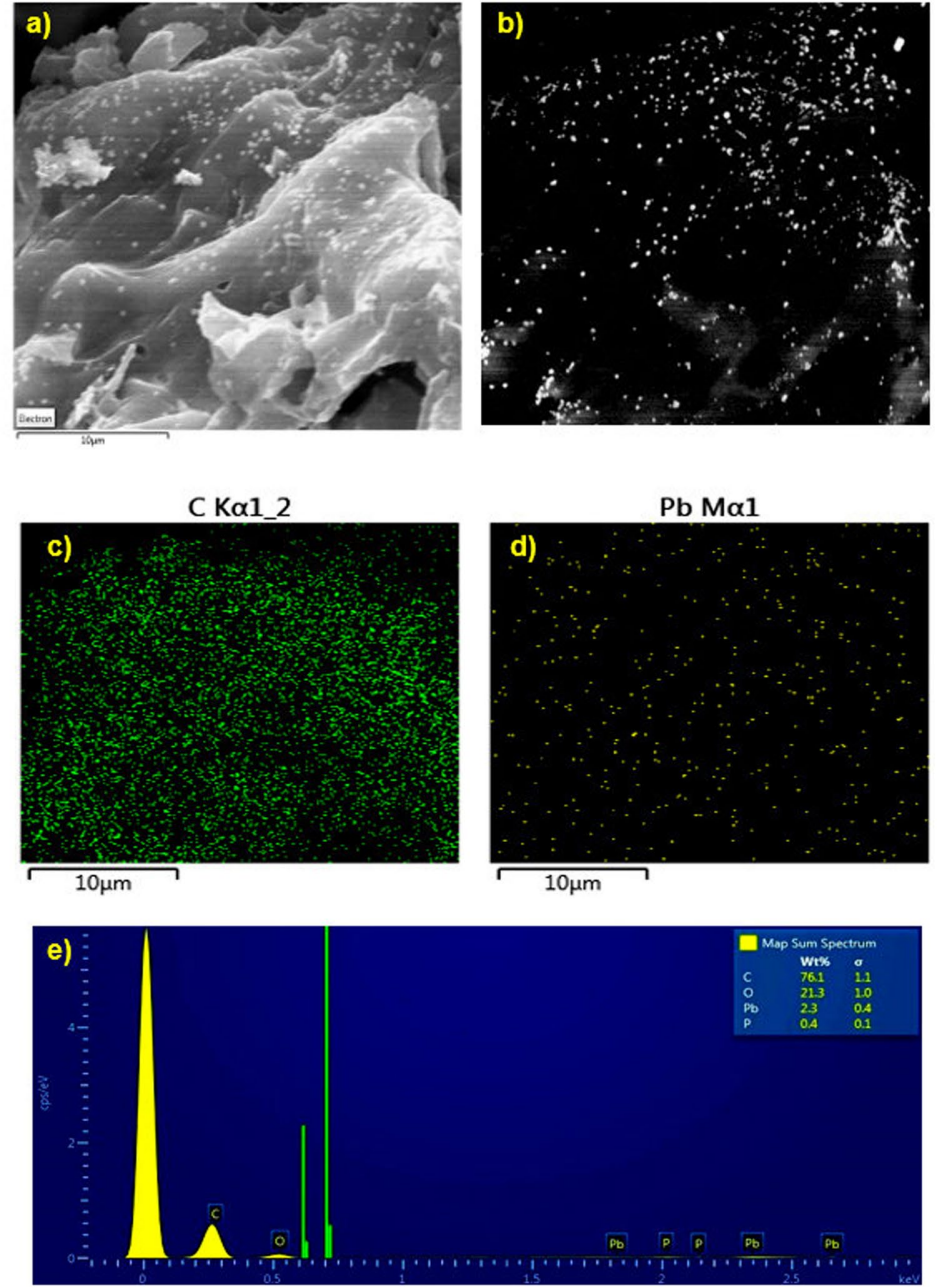
SEM- EDX analysis of lead activated carbon (**a**) ETD image (**b**) BSED image, (**c**) carbon map, (**d**) lead map and (**e**) EDX results.

The SEM/EDX of the lead activated carbon (AC-K spent sample) along with the elemental mapping of Pb and C as shown in Fig. 8. The SEM images using ETD and BSED are shown in Fig. 8(a,b), respectively, where Pb is shown in brighter spots due to its heavier atomic weight compared to carbon as shown in Fig. 8(b). The elemental mapping (Fig. 8(c,d) show that carbon is dominating the surface of the activated carbon (AC-K) compared to the lead metal. The EDX result (Fig. 8(e) shows the wt.% composition of the lead AC-K with 76.1, 21.3 and 2.3 wt.% for C, O and Pb, respectively.

If one is to imply the practice of sustainability to low-value biomass (such as miscanthus), it has to be in the form of action and not just conceptualisation through words. Low-value biomass (miscanthus) is not just an environmental and combustion problem, but a loss to our economy when considering the promising transformation to activated carbon and carbon nanotubes, such as the ones developed through the multi-activation steps herein. These are considered as value-added materials that possess a wide range of properties and have application in areas such as wastewater remediation, micro-electronics, smart & novel composites and bone growth in tissue engineering. Viewing miscanthus in this way provides sustainable biomass management options^41^ and directly supports the views of Whetten^42^, in that this study can be considered as a forward-thinking & expansive step and building block in the theoretical aspects of carbon nanotubes and their synthesis to create a value-added product with enhanced roles and properties to their precursor counterpart. Opening these avenues for an otherwise problematic waste stream with limited utilisation options directly follows the mantra of sustainability and provides a foreground for these applications to be tested further in other studies. This provides several end uses to this low-value stream, all of which are considered up-cycling & valorisation approaches and directly supporting and facilitating the concept of the circular economy, whilst also being considered as a route to cleaner production of these materials^9^.

The significance of this work is that in our local demographic of Northern Ireland there is an abundance of miscanthus waste. As we have focused on miscanthus and its physicochemical properties before in the past, we have also noticed its high silicon content which hinders it useless for thermochemical applications. Due to this, there is a need to be able to utilize this waste biomass in a different way, one in which it can serve a purpose after it has been rendered useless. Using this material for activated carbon or carbon nanotubes helps alleviate the waste problem here locally, whilst also allowing these novel materials to be used elsewhere. As both AC and CNTs are widely sought after, this study can help produce these in a cost-effective way. Our novel method which was outlined earlier in a previous study was applied to this problematic waste material and the results have been extremely positive in that, miscanthus AC and CNTs have proved to be far superior to other biomass counterparts such as potato peel waste and barley waste.

Waste *Miscanthus* × *giganteus* was used in this study for the synthesis of activated carbon via two activation steps, firstly with phosphoric acid (AC-P) which produced a porous carbon with a surface area of 1142 m^2^.g^−1^ and a pore volume of 0.70 cm^3^.g^−1^. Secondly, AC-P was further treated with a KOH activation step to produce a better AC-K with a surface area of 1368 m^2^.g^−1^ and a pore volume of 0.92 cm^3^.g^−1^. Finally, CNTs were synthesized from the produced AC-K and found to be a superhydrophilic multi-wall CNTs type with a contact angle of θ = 9.88°. AC-P, AC-K and CNTs were used in heavy metal removal (HMR) with the potential removal of lead up to 90% in the first hour. AC-K was the optimum material in this study utilized in HMR, making it an ideal candidate in rapid removal of heavy metals in wastewater treatment or in adsorption applications. This proposed route of synthesising high surface area AC and superhydrophilic multi-walled CNTs from this waste stream helps contribute to the circular economy by up-cycling an otherwise waste and problematic thermochemical conversion feedstock by adding value and other potential routes for application such as wastewater treatment and other end uses for AC and CNTs.

## Materials and Methods

The miscanthus plant was harvested from a 10-yr-old energy crop grown at the Agri-Food and Biosciences Institute (AFBI), Environment & Renewable Energy Centre, Hillsborough, Northern Ireland (54.453077, −6.086162)^43^. Followed by drying at ~110 °C until completely dry, then crushed into a form of powder and sieved to obtain particles of 110- 300 µm size for the pyrolysis process.

### The preparation method for activated carbon and carbon nanotubes materials

The preparation method for activated carbon and carbon nanotubes is described elsewhere^44^. Prior to the chemical activation method, miscanthus raw biomass was dried and crushed as described above. The first chemical activation method was achieved by using phosphoric acid to open the pores of the raw miscanthus biomass through the formation of C-O-P bonding on the surface of miscanthus. This was performed by mixing 11.4 g of miscanthus with 11.87 mL of orthophosphoric acid (85 wt.% in H_2_O, 99.99% trace metals basis, Sigma-Aldrich, UK) along with 150 mL of deionized water. Followed by stirring the mixture at 86 °C for 3 hrs using a hot plate, then drying in the oven at 110 °C, then the pyrolysis at 500 °C (2 °C.min^−1^) under N_2_ atmosphere with a flow rate of 100 mL.min^−1^. After reaching a temperature of 500 °C and maintained for 0.5 hr, cooling down the sample and washing the produced AC with deionised water till neutralisation at a pH of 7, then drying at 110 °C for one day. The produced AC material was designated as AC-P.

The second chemical activation method was achieved using potassium hydroxide, by mixing AC-P: KOH with a weight ratio of 1:3.5 (wt/wt%) with deionised water. Followed by stirring at 100 °C for 1 hr, drying overnight at 110 °C then pyrolysis as in the first activation method at 500 °C. To remove the excess of KOH from the produced AC material, hydrochloric acid (Sigma Aldrich UK, reagent grade, 37%) was used to wash the material till neutralisation at pH of 7, followed by drying for one day at 110 °C, then the produced AC designated as AC-K.

Finally, the carbon nanotubes material was prepared by mixing 1 g of AC-K with 17.5 g of melamine (2, 4, 6-triamino-1,3,5-triazine, 99%, Sigma-Aldrich, UK) and 0.5 g of iron oxalate (Fe_2_(C_2_O_4_)_3_ 6H_2_O, Sigma-Aldrich, UK) and mixed in methanol (HPLC grade, ≥99.9%, Sigma-Aldrich, UK). The mixture was stirred at room temperature, then washing with deionised water before drying overnight and then subjected to two-stage pyrolysis under N_2_ atmosphere where the temperature ramped to 600 °C (2 °C.min^−1^) and then held for 3 hrs before being subsequently pyrolysed to 900 °C (2 °C.min^−1^) and held for an hour, then cooled down to room temperature. The produced CNTs was then washed several times before drying at 110 °C overnight. The final CNTs produced was designated as CNTs.

### Lignocellulosic biomass Characterization

The raw biomass along with the produced ACs and CNTs were characterised using XRD, SEM-EDX, contact angle meter, CHNS, TGA/DSC and FTIR techniques. The details of those techniques are provided in the Supplementary Information (ESI).

## Supplementary information

Supplementary Information.
