# Supplementary material for: The production and application of carbon nanomaterials from high alkali silicate herbaceous biomass
Source: Sci Rep. 2020 Feb 13;10:2563. doi: 10.1038/s41598-020-59481-7 (PMC7018825; doi:10.1038/s41598-020-59481-7)
Supplement: Supplementary file 1 — Supplementary Information. [file 41598_2020_59481_MOESM1_ESM.docx]

*Supplementary Information of*

**The production and application of carbon nanomaterials from high alkali silicate herbaceous biomass**

**Ahmed I. Osman ^a,b*^, Charlie Farrell ^c,d^, Ala'a H. Al-Muhtaseb^e*^, John Harrison ^c^_,_ David Rooney ^a^**

^a^ School of Chemistry and Chemical Engineering, Queen’s University Belfast, Belfast BT9 5AG, Northern Ireland, UK.

^b^ Chemistry Department, Faculty of Science - Qena, South Valley University, Qena 83523 – Egypt.

^c^ South West College, Cookstown, Co. Tyrone, BT80 8DN, Northern Ireland, UK.

^d^ School of Mechanical and Aerospace Engineering, Queen’s University Belfast, Belfast BT9 5AH, Northern Ireland, UK.

^e^ Department of Petroleum and Chemical Engineering, College of Engineering, Sultan Qaboos University, Muscat, Oman.

Corresponding Author: Ahmed Osman, Ala’a H. Al-Muhtaseb

Email: [aosmanahmed01@qub.ac.uk](mailto:aosmanahmed01@qub.ac.uk), muhtaseb@squ.edu.om

Address: School of Chemistry and Chemical Engineering, Queen's University Belfast, David Keir Building, Stranmillis Road, Belfast BT9 5AG, Northern Ireland, United Kingdom

Fax: +44 2890 97 4687

Tel.: +44 2890 97 4412

*Characterization techniques:*

Powder X-ray diffraction (XRD) was carried out using a PANalytical X’Pert Pro X-ray diffractometer. This diffractometer was equipped with a CuK_α_ X-ray source with a wavelength of 1.5405 Ǻ. The diffractograms were collected up to 2θ = 80°. The X-ray tube was set at 40 kV and 40 mA.

Brunauer-Emmett-Teller (BET) analysis was performed using a Micromeritics ASAP 2020 system. BET surface area and pore volume were measured by N_2_ adsorption and desorption isotherms at liquid nitrogen temperature (-196 °C).

Scanning Electron Microscopy (SEM) was carried out on a FEI Quanta 250 FEG MKII with a high-resolution environmental microscope (ESEM) using XT Microscope Control software and linked to an energy-dispersive X-ray (EDX) detector. Two types of detectors were used in SEM analysis; the Everhart-Thornley Detector (ETD) which is used to detect secondary electrons emitted from the sample and Back-Scattered Electron Detector (BSED). The EDX used was a 10 mm^2^ silicon drift detector (SDD)-x-act from Oxford Instruments which utilizes Aztec® EDS analysis software. Both systems used the same chamber.

The static contact angle of the catalyst pellets with water was measured using a contact angle meter equipped with a CCD camera (FTA1000 Drop Shape Instrument- B Frame system).

The composition of the miscanthus sample was characterised by means of proximate and ultimate analyses. Elemental (C, H and N) Analysis was performed using a Perkin Elmer PE2400 CHNS/O Elemental Analyzer. The oxygen content was calculated by difference from the data obtained by a Perkin Elmer PE2400 CHNS/O Elemental Analyzer machine.

TGA was performed from 50 to 800-1000 °C with different heating rates of 2, 5, 15, 25 and 50 °C.min^-1^, in a stream of dry N_2_ flowing at 20 cm^3^.min^-1^, using a simultaneous thermal analysis Mettler Toledo (TGA/DSC) Thermogravimetric analyzer Pyris TGA/DSC1. Changes in mass of the sample were recorded during the ramping operation. Differential Scanning Calorimetry ( DSC) was used to determine the heat liberated in watts per gram.

Fourier Transform Infrared (FT-IR) spectroscopy was operated using a Perkin Elmer Spectrum, a beam splitter in the wavenumber range of 4000 – 400 cm^-1^.

**
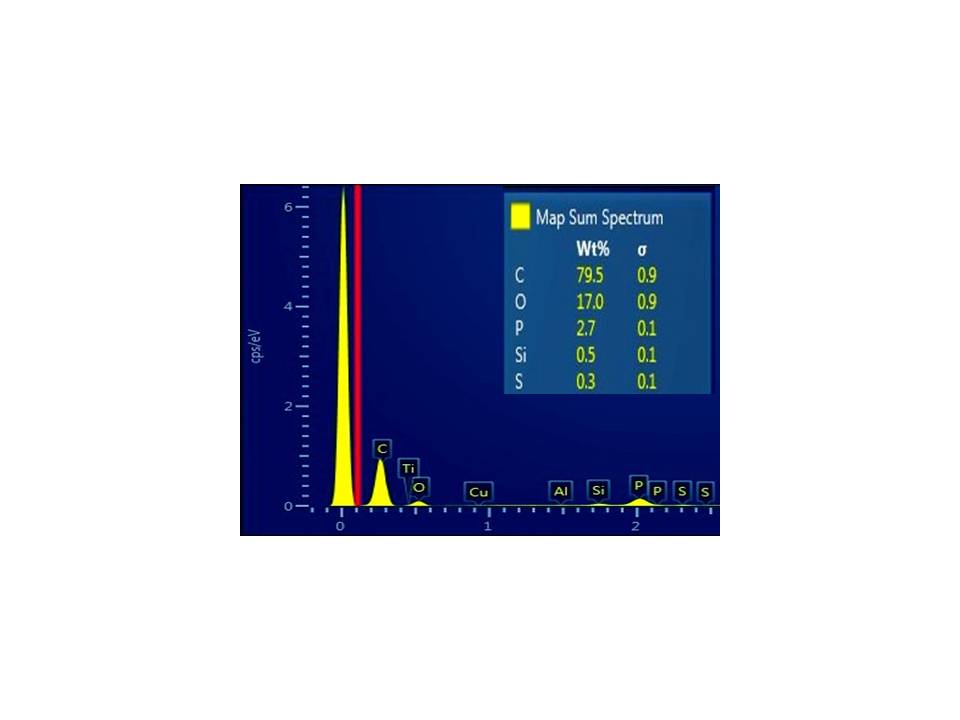
**

**a)**

**
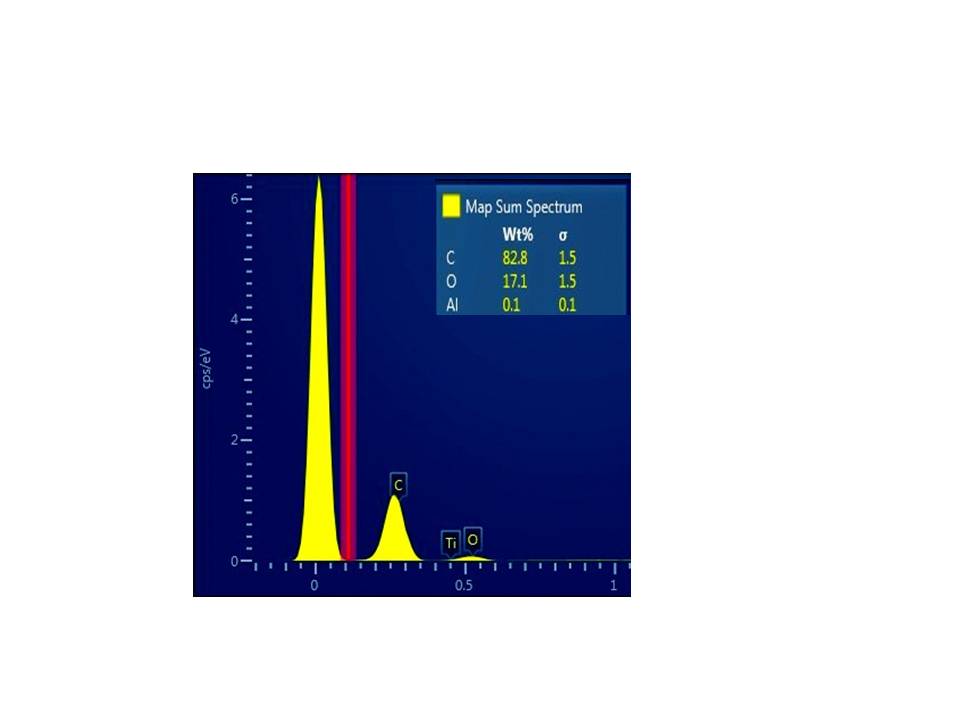
**

**b)**

**
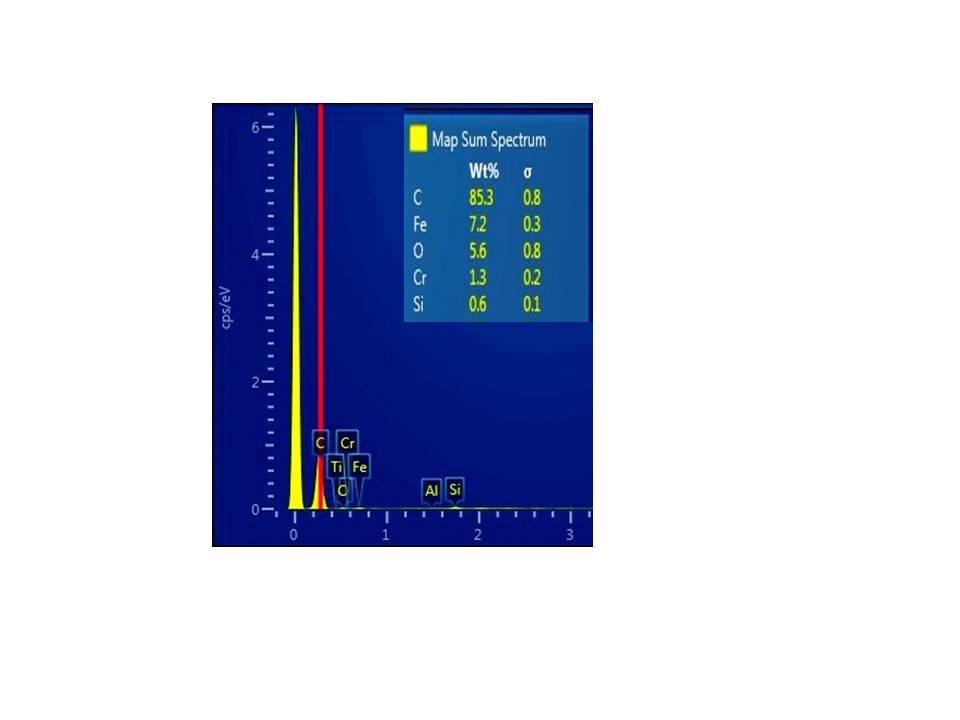
**

**c)**

**Figure S1:** SEM images for a) H_3_PO_4_ activation, b) KOH activation and c) CNTs at a different level of magnifications using ETD detector.

**Figure S2:** TGA curves of CNTs under N_2_ atmosphere (20 ml.min^-1^).

**a)**

**e)**

**d)**

**g)**

**Figure S3:** XPS of miscanthus activated carbon (dash line) and CNTs (solid line) of a) C*1s*, b) O*1s*, C) N*1s*, d) Si*2p*, e) P*2p*, f) Fe*2p* and g) XPS survey.
